# Circulating mRNAs and miRNAs as candidate markers for the diagnosis and prognosis of prostate cancer

**DOI:** 10.1371/journal.pone.0184094

**Published:** 2017-09-14

**Authors:** Marilesia Ferreira de Souza, Hellen Kuasne, Mateus de Camargo Barros-Filho, Heloísa Lizotti Cilião, Fabio Albuquerque Marchi, Paulo Emilio Fuganti, Alexandre Rossi Paschoal, Silvia Regina Rogatto, Ilce Mara de Syllos Cólus

**Affiliations:** 1 General Biology Department, State University of Londrina, Londrina, Paraná, Brazil; 2 CIPE, AC Camargo Cancer Center, São Paulo, São Paulo, Brazil; 3 Londrina Cancer Hospital, Londrina, Paraná, Brazil; 4 Department of Computing, Federal University of Technology—Paraná, UTFPR, Cornélio Procópio, Paraná, Brazil; 5 Department of Clinical Genetics, Vejle Hospital and Institute of Regional Health Research, University of Southern Denmark, Vejle, Denmark; University of South Alabama Mitchell Cancer Institute, UNITED STATES

## Abstract

Circulating nucleic acids are found in free form in body fluids and may serve as minimally invasive tools for cancer diagnosis and prognosis. Only a few studies have investigated the potential application of circulating mRNAs and microRNAs (miRNAs) in prostate cancer (PCa). The Cancer Genome Atlas (TCGA) database was used for an *in silico* analysis to identify circulating mRNA and miRNA as potential markers of PCa. A total of 2,267 genes and 49 miRNAs were differentially expressed between normal and tumor samples. The prediction analyses of target genes and integrative analysis of mRNA and miRNA expression revealed eleven genes and eight miRNAs which were validated by RT-qPCR in plasma samples from 102 untreated PCa patients and 50 cancer-free individuals. Two genes, *OR51E2* and *SIM2*, and two miRNAs, miR-200c and miR-200b, showed significant association with PCa. Expression levels of these transcripts distinguished PCa patients from controls (67% sensitivity and 75% specificity). PCa patients and controls with prostate-specific antigen (PSA) ≤ 4.0 ng/mL were discriminated based on *OR51E2* and *SIM2* expression levels. The miR-200c expression showed association with Gleason score and miR-200b, with bone metastasis, bilateral tumor, and PSA > 10.0 ng/mL. The combination of circulating mRNA and miRNA was useful for the diagnosis and prognosis of PCa.

## Introduction

Prostate cancer (PCa), the most frequently diagnosed neoplasia of solid organ among men in Brazil and the second most common cancer worldwide, is the fifth leading cause of death by cancer in the world [1,2]. Digital rectal examination and prostate-specific antigen (PSA) test are widely used for screening of PCa. However, these methods lack the efficiency for the detection of all tumor types and differentiation between advanced and latent disease forms. In addition, the PSA test exhibits low specificity (37%) [3] and may lead to overdiagnosis in patients with non-neoplastic prostate diseases [4]. As a result, several patients are submitted to unnecessary biopsy every year [5].

In the USA, more than 1 million men were diagnosed with this disease and received unnecessary treatment since PSA introduction [6]. Long-term studies have shown that the PSA test either caused only a moderate reduction in mortality or was insignificant [7–9]. This evidence resulted in the non-recommendation of the use of PSA test for screening PCa in USA [10], thereby necessitating new biomarkers for better diagnosis and prognosis of PCa [1,11]. However, PSA test can be used with caution until new markers are introduced in clinical routine [12,13].

Circulating nucleic acids (cfNAs) are free DNA and RNA molecules in the plasma, serum, and urine of cancer patients and healthy individuals, which can be used as minimally invasive diagnostic tools [14]. These molecules are found in body fluids in free forms or bound to proteins or exosome-associated. cfNAs originate from necrotic and apoptotic cells or are secreted by several cell types [14,15]. These molecules are stable and exhibit potential to be used as biomarkers; however, a limited number of studies have focused on circulating RNAs (cfRNAs) in PCa [16,17]. cfNAs may be served as a "liquid biopsy," which would be useful for diagnostic applications without the need for biopsy. Furthermore, these molecules are powerful tools for monitoring the disease and to evaluate the efficacy of treatment in a rapid non-invasive technique [14].

Among cfRNAs, the circulating microRNAs (cfmiRNAs) have been intensively studied [15]. miRNAs are small non-coding RNA molecules of approximately 22 nucleotides long; acting as post-transcriptional regulators and exhibiting preferential binding to the 3′ untranslated region (3'UTR) of mRNAs [18]. A few studies have focused on cfmiRNAs as new attractive cancer biomarkers, with miR-141 and miR-375 being the most promising miRNAs described in prostate cancer [19,20]. However, the role of these aberrant circulating miRNAs is unclear. Recently, Wang et al. [21] reported the involvement of the miR-410-5p in the modulation of the communication between cancer cells and dendritic cells. Previously, the authors described its potential as serum diagnostic marker in prostate cancer [22]. Nevertheless, circulating mRNAs (cfmRNAs) have been poorly explored in cancer research [14]. In prostate cancer, the genes telomerase reverse transcriptase (*hTERT*) [23] and bone morphogenetic protein-6-specific (*BMP6*) [24] are reported as biomarkers for PCa diagnosis.

In this study, the Cancer Genome Atlas (TCGA) database was used for the identification of mRNA and miRNAs as potential diagnostic and prognostic markers for PCa. Plasma samples from untreated patients with PCa and cancer-free control subjects were evaluated for the selected genes and miRNAs. Two genes and two miRNAs differentiated cancer patients from controls with sensitivity and specificity higher than the PSA test. miRNAs expression levels were identified as potential markers for aggressive disease. Our data provide an additional support for the potential use of cfmRNAs and cfmiRNAs as PCa markers.

## Material and methods

### Patients

From 2014 to 2015, a total of 102 patients were enrolled at the Londrina Cancer Hospital (Londrina-PR, Brazil). These patients were diagnosed with PCa and failed to receive any treatment before sample collection. Fifty hospital-based cancer-free individuals without urinary disease symptoms and PSA level ≤ 4.0 ng/mL were included as controls. Ethnic groups were categorized based on the ethnic-racial classification by the Brazilian Institute of Geography and Statistics [25]. Individuals who never smoked or quit smoking for 10 years or more were considered non-smokers, while those who never consumed alcohol or quit alcohol for 10 years or more were considered non-alcoholics. Clinical and histopathological data were obtained from the available medical and pathological reports, respectively. The study was approved by the Research Ethics Committee of the State University of Londrina (CAAE19769913.0.0000.5231). All participants provided written informed consent and answered a modified questionnaire based on Carrano and Natarajan [26]. Epidemiological and clinical characteristics of all participants are shown in Table 1.

**Table 1:** Epidemiological and clinical characteristics of patients with prostate cancer and control subjects.

Characteristics		Patients	Controls	*P* value
		N (%)	N (%)	
Age (years)	< 65	30 (29.4)	26 (52.0)	0.007*
	≥ 65	72 (70.6)	24 (48.0)	
Ancestry	Caucasian	79 (77.5)	39 (78.0)	0.94
	African^a^	23 (22.5)	11 (22.0)	
Smoking habit	Yes	31 (30.4)	15 (30.0)	0.96
	No	71 (69.6)	35 (70.0)	
Alcohol consumption	Yes	58 (56.9)	31 (62.0)	0.55
	No	44 (43.1)	19 (38.0)	
Exposure to xenobiotic	Yes, agrochemicals	54 (52.9)	24 (48.0)	0.68
	Yes, others	22 (21.6)	14 (28.0)	
	No	26 (25.5)	12 (24.0)	
Family history of cancer	Yes	62 (60.8)	23 (46.0)	0.09
	*Yes*, *prostate*	16 (15.7)	4 (16.0)	
	No	40 (39.2)	27 (54.0)	
PSA^b^^ ^(ng/mL)	≤ 4.0	9 (8.8)	50 (100.0)	
	4.1 to 10.0	39 (38.2)		
	> 10.0	54 (52.9)		
Gleason score	3 to 6	58 (56.9)		
	7	32 (31.4)		
	8 to 9	10 (9.8)		
	Missing	2 (1.9)		
Bilateral tumor	P	47 (46.1)		
	A	52 (51.0)		
	Missing	3 (2.9)		
Bone metastasis	P	11 (10.8)		
	A	88 (86.3)		
	Missing	3 (2.9)		
Treatment^c^^,^^d^	Prostatectomy	61 (59.8)		
	Hormone therapy	16 (15.7)		
	Radiotherapy	19 (18.6)		
	Orchiectomy	8 (7.8)		
	Others^e^	10 (9.8)		

^a^Afro-descendants and black

^b^PSA = Prostate-specific antigen

^c^Treatment performed after collection

^d^Patients may receive one or more treatment types

^e^Patients who are under active surveillance or refused treatment

P = Present; A = Absent.

*Statistically significant difference (Student’s t-test, P < 0.05)

### Selection of miRNAs and candidate genes

Candidate miRNAs and mRNAs were selected *in silico* using gene expression and miRNA data available on TCGA data portal (https://tcga-data.nci.nih.gov). The expression data of miRNA (miRNA-seq) and mRNA (RNAseqV2) were obtained using the Illumina HiSeq platform considering level 3. Data from 425 PCa tissue samples and 52 surrounding normal tissue (SNT) samples were analyzed. The differentially expressed transcripts were selected based on the following parameters: fold change (FC) > 2, adjusted *P* < 0.001, and false discovery rate (FDR) < 0.001. We used four strategies to define candidate genes and miRNAs as follows: i) integration analyses of mRNA and miRNA data and prediction of target genes using miRWalk [27] and miRTarBase [28] databases; ii) analysis of clinical (PSA) and histopathological features (lymph node invasion, Gleason score, tumor stage) of these samples with *P* < 0.05; iii) gross number of reads obtained from the sequencing data, considering values greater than 1,000; and iv) investigation in the literature of the candidate mRNAs and miRNAs in prostate cancer. A comparison analyses between groups were performed using the two-sample *t*-test with the BRB ArrayTools software [29]. The genes and miRNA selected for validation in plasma samples were tested *in vitro* to assess the secretion by prostate cancer cells.

### Sample collection and circulating RNA extraction

Peripheral blood samples were collected through intravenous infusion with needles and disposable BD Vacutainer^®^ tubes containing 6% ethylenediaminetetraacetic acid (EDTA) from all individuals. Blood samples were placed on ice and processed within 2 hours after collection. The whole blood was centrifuged at 700 x*g* for 10 minutes. To avoid cellular contamination, enrichment of cfNAs was performed following the protocol described by Duttagupta et al. [30]. Blood plasma was subjected to centrifugation at 2,000 x*g* for 10 minutes at 4°C. Following centrifugation, the cell-free plasma was stored at -80°C until use.

Extraction of total cfRNAs was performed using the miRNeasy Mini kit (Qiagen, Hilden, Germany) with some modifications to the manufacturer’s protocol. Briefly, 1 mL of plasma sample was divided into five tubes each containing 200 μL of plasma. Each tube was treated with 1 mL TRIzol™ reagent (Thermo Fisher Scientific), vortexed for 1 minute, and incubated at room temperature for 5 minutes. Following incubation, the mixture was treated with 200 μL chloroform and vortexed for 15 seconds. The solution was incubated at room temperature for 3 minutes, followed by centrifugation at 12,000 x*g* for 15 minutes at 4°C. The supernatant was transferred to a fresh tube and homogenized using 1.25 volumes of 100% ethanol. A total of 700 μL of the solution was transferred to a binding column with a collection tube and centrifuged at 8,000 x*g* for 15 seconds. The flow-through was discarded and the process repeated for about 12 times. After column saturation, the column was washed, and the sample eluted using 25 μL RNase-free water. Samples were quantified using NanoDrop 2000 spectrophotometer (Thermo Fisher Scientific, Wilmington, DE, USA).

### Analysis of mRNA and miRNA expression with quantitative reverse transcription PCR (RT-qPCR)

The expression level of the selected genes was determined by qPCR. Briefly, 500 ng of total RNA sample was used for the reverse transcriptase (RT) reaction using oligo-DT, random primers, and 60 Superscript III units (Invitrogen, Carlsbad, CA, USA) following the manufacturer’s protocol. Each reaction contained 5 μL of Sso Advanced Universal SYBR Green Supermix (Bio-Rad, USA), 5 μM of each primer, and 10 ng of cDNA. The reaction was performed on the 7900HT Fast Real-Time PCR System Thermocycler (Applied Biosystems, Singapore). Primers were obtained from KiCqStart® SYBR Green Primers (Sigma-Aldrich, Saint Louis, MO, USA) (S1 Table). Transcript analyses were performed using the ABI Prism 7900 Sequence Detection System (Applied Biosystems, Singapore) software. The quality of amplification product was verified by the analysis of the dissociation curve.

Expression patterns of miRNAs were performed using 5 ng of total cfRNA and TaqMan miRNA Reverse Transcription kit (Applied Biosystems, Foster City, CA, USA) following the manufacturer’s instruction. Each reaction was eluted at a 1:4 ratio and contained 5.5 μL of TaqMan 2X Universal PCR Master Mix (Applied Biosystems, Wootston Warrington, WA, UK), 0.45 μL of miRNA-specific TaqMan Probe (Applied Biosystems, Foster City, CA, USA), and 7 μL cDNA of the diluted RT reaction. The reaction was performed on the 7900HT Fast Real-Time PCR System Thermocycler. The reaction was assembled with robot pipetting using QIAgility (QIAGEN, Courtaboeuf, France) in duplicate. Although there is no consensus on the selection of optimal endogenous controls for cell free DNA, we combined two genes (GAPDH and ACTB) and miRNAs (RNU6B and RNU48) widely used as controls [31–36]. A pool of samples was used as calibrator.

### Statistical analysis

The amplification efficiency for the pre-designed assays (TaqMan Probe for miRNA and KiCqStart® SYBR Green Primers for mRNA) is estimated as “essentially” as 100%. The mathematical model of 2-ΔΔct was applied to obtain the relative expression data [37]. Levene test and Student's *t*-test were used to evaluate the sample distribution and compare means between groups, respectively (GraphPad Prism Software version 5.00, San Diego, California, USA). Descriptive analysis was performed using the IBM SPSS Statistics 22.0 software (IBM Corp., Armonk, New York, USA). The Receiver Operating Characteristic (ROC) curve was used to evaluate the diagnostic test ability (specificity, sensitivity and cut-off points of each marker). The area under the curve is a measure of the discriminant power of a diagnostic test. The ROC curves were constructed using the MedCalc Statistical Software version 16.8.4 (MedCalc Software bvba, Ostend, Belgium). A value of *P* < 0.05 was considered statistically significant.

A score was developed for joint analysis of the markers. For each gene, an optimal cut-off point was determined through ROC curve analyses. One point was assigned to the individual who presented levels of expression superior to the cut-off point. The final score was determined by the sum of the points, with a maximum possible score of four points in a blind test. Individuals with three or more points were considered positive for PCa.

## Results

### Selection of miRNAs and mRNA candidate

**Fig 1:**
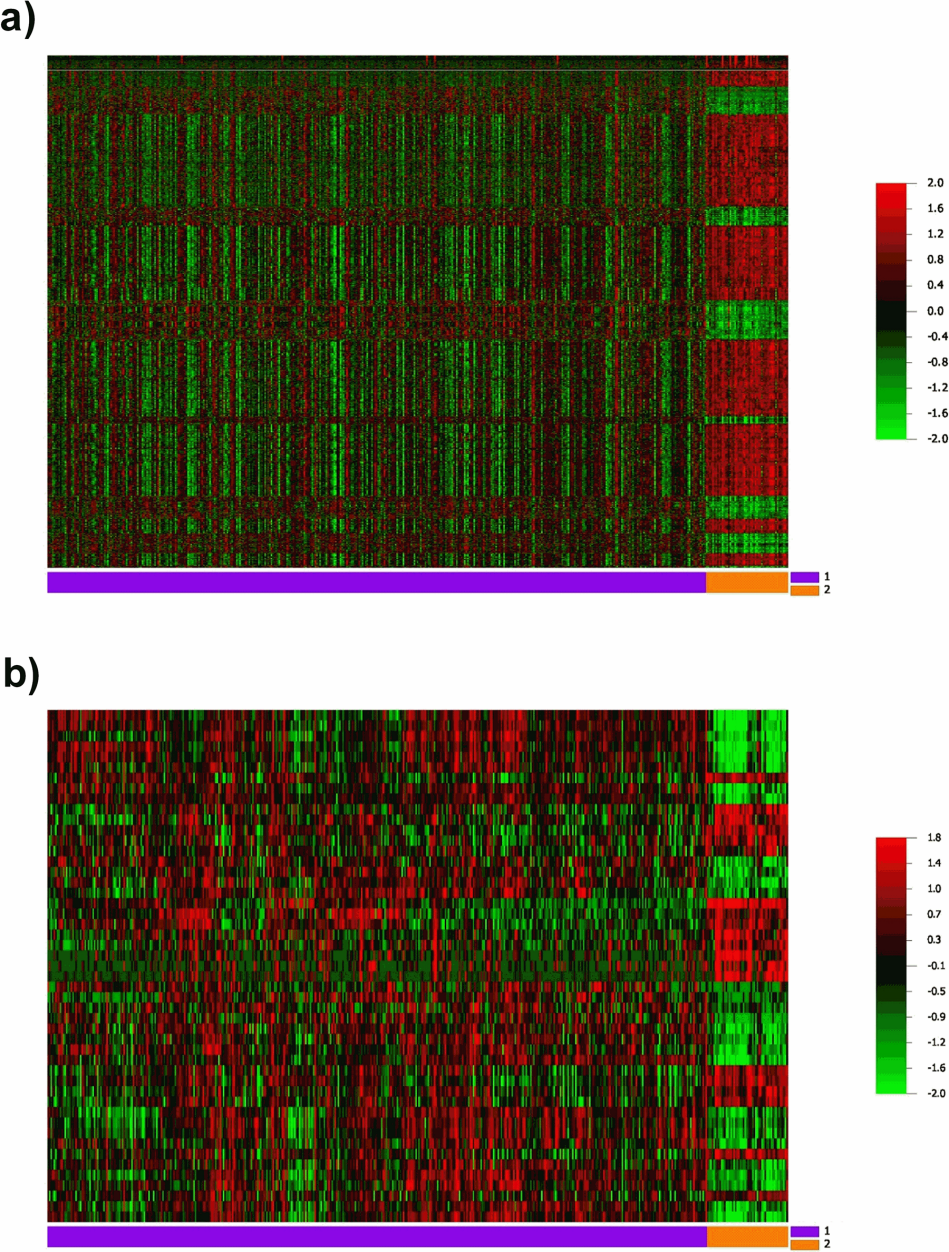
Heatmap showing gene and miRNA differential expression in tumor tissue samples (1: purple) compared with surrounding normal tissues (2: orange) from TCGA data. **a** Global gene expression analysis revealed 2,267 differentially expressed genes (P ≤ 0.001 and FDR ≤ 0.001); **b** miRNA expression analysis revealed 49 miRNAs differentially expressed (P ≤ 0.001 and FDR ≤ 0.001). Each line in the left axis represents a gene/miRNA. Columns represent each sample.

**Fig 2:**
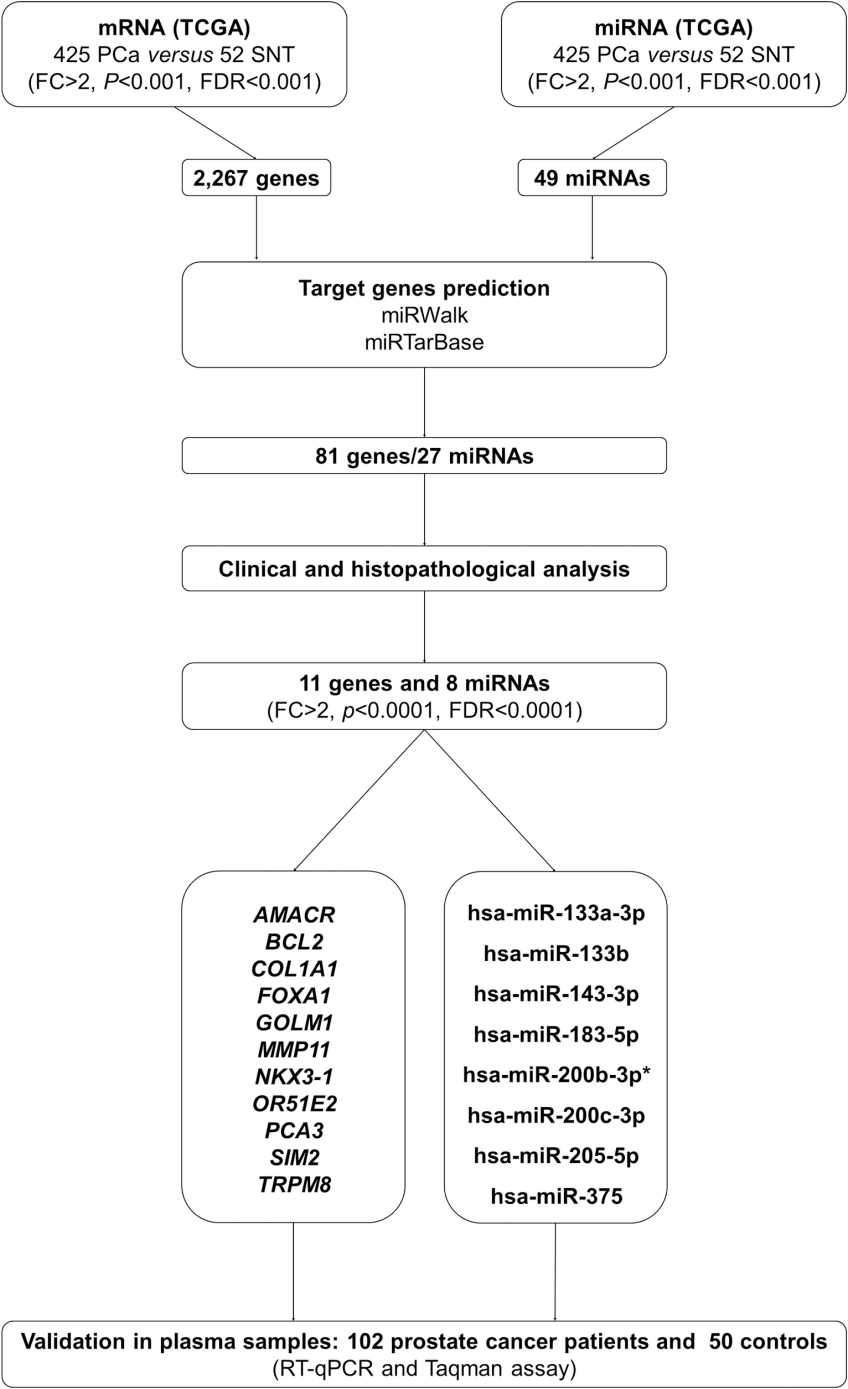
Flowchart of the analyses to define candidate genes. *Gene selected from Pubmed database.

The analysis of TCGA expression data for transcripts from the 425 PCa and 52 SNT samples is shown in Fig 1. A clear difference in the mRNA and miRNA expression profiles was observed between the groups analyzed. Our results revealed a differential expression of 2,267 genes and 49 miRNAs between tumor and normal tissues (FC > 2, *P* < 0.001, FDR < 0.001) (Fig 1, S2 and S3 Tables). The prediction analyses of target genes, followed by the integrative analysis of the mRNA and miRNA expression data were performed. Only samples with results of both analyses (mRNA and miRNA) were included. An inverse correlation was detected between the expression levels of 81 target genes and 27 differentially expressed miRNAs. To perform gene and miRNA assortment, clinical and histopathological characteristics and the number of reads obtained from the sequencing data were considered. Seven miRNAs (hsa-miR-143-3p, hsa-miR-183-5p, hsa-miR-200c-3p, hsa-miR-375, hsa-miR-133b, hsa-miR-205-5p, and hsa-miR-133a-3p) were chosen. Additionally, the miR-200b-3p, described as involved in the prostate cancer cell proliferation and metastasis and associated with prognosis [38–40] was selected. Eight miRNAs and 11 genes (*AMACR*, *BCL2*, *COL1A1*, *FOXA1*, *GOLM1*, *MMP11*, *OR51E2*, *NKX3-1*, *PCA3*, *SIM2*, and *TRPM8*) obtained, were evaluated in plasma samples by RT-qPCR (Fig 2 and Table 2).

**Table 2:** Summary of miRNAs and their targets predicted from in silico analyses based on TCGA database.

MicroRNA	Fold-change^a^	Predicted target genes
hsa-miR-133a-3p	0.28	*AMACR*, *BCL2*, *COL1A1*, *NKX3-1*, *SIM2*
hsa-miR-133b	0.27	*AMACR*, *BCL2*, *COL1A1*, *NKX3-1*, *SIM2*
hsa-miR-143-3p	0.40	*AMACR*, *BCL2*, *COL1A1*, *GOLM1*, *MPM11*, *NKX3-1*, *OR51E2*, *SIM2*
hsa-miR-183-5p	4.35	*AMACR*, *COL1A1*, *FOXA1*, *MPM11*, *NKX3-1*, *TRPM8*
hsa-miR-200b-3b^b^	-	*AMACR*, *BCL2*, *GOLM1*, *OR51E2*, *SIM2*, *TRPM*
hsa-miR-200c-3p	3.33	*AMACR*, *BCL2*, *FOXA1*, *GOLM1*, *OR51E2*, *SIM2*, *TRPM8*
hsa-miR-205-5p	0.36	*AMACR*, *BCL2*, *COL1A1*, *GOLM1*, *MPM11*, *NKX3-1*, *SIM2*, *TRPM8*
hsa-miR-375	6.74	*AMACR*, *BCL2*, *COL1A1*, *FOXA1*, *GOLM1*, *NKX3-1*, *SIM2*, *TRPM8*

^a^Fold-change based on The Cancer Genome Atlas (TCGA) data; tumor tissue *versus* normal tissue.

^b^miRNA selected after investigation in the literature of the candidate miRNAs in prostate cancer

### *OR51E2*, *SIM2*, miR-200c, and miR-200b are potential circulating diagnostic markers of PCa

Among the 11 genes investigated with qPCR, three (*COL1A1*, *FOXA1*, and *MMP11)* were excluded based on the quality criteria of reactions and six (*AMACR*, *BCL2*, *GOLM1*, *NKX3-1*, *PCA3*, and *TRPM8*) displayed no differences between PCa and controls samples. In comparison to the control subjects, patients with PCa exhibited differential expression of *OR51E2* (FC = 6.98, *P* = 0.002, area under the curve [AUC] = 0.65) and *SIM2* (FC = 1.9, *P* = 0.02, AUC = 0.61) genes.

**Fig 3:**
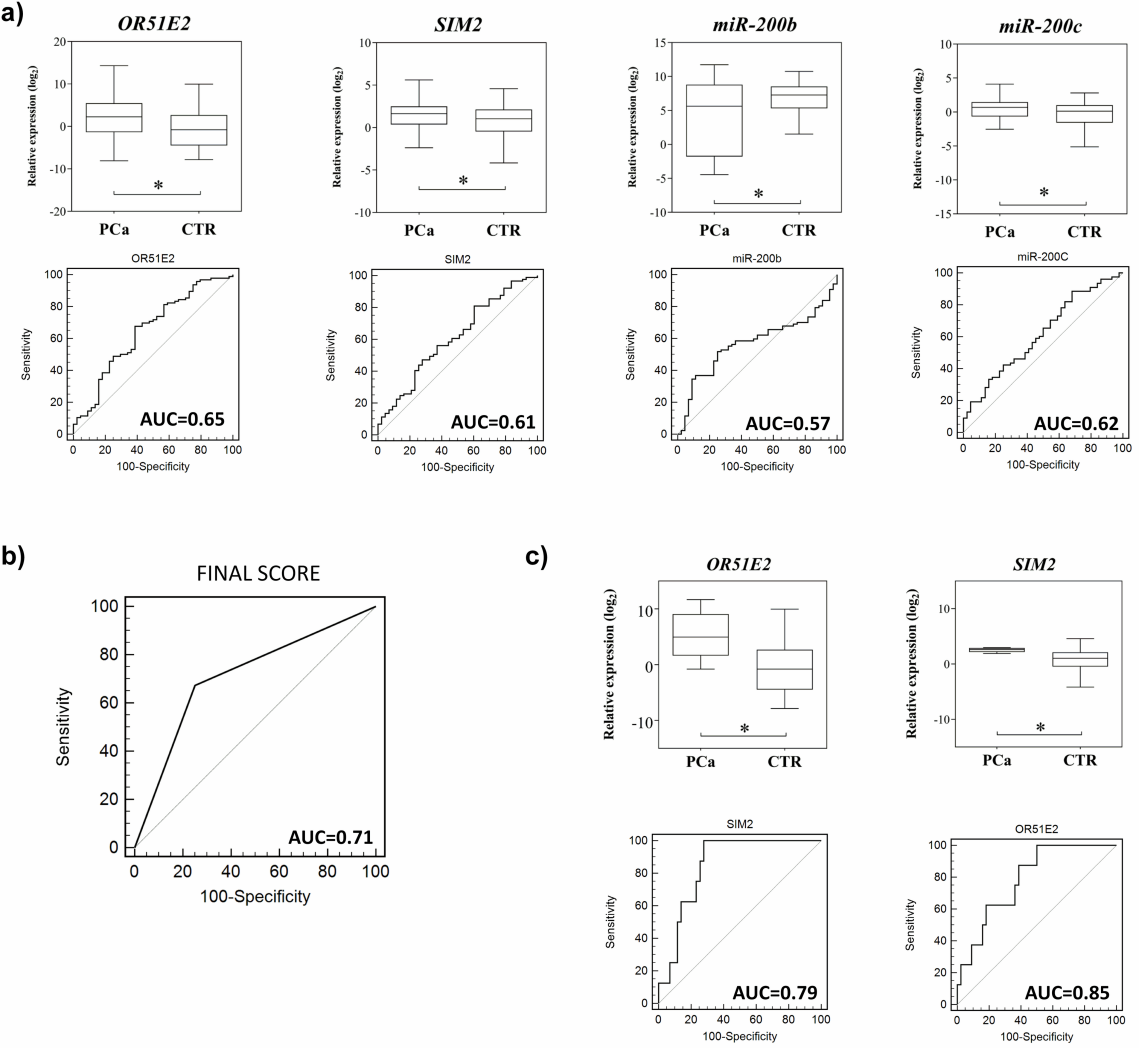
Differential expression of circulating mRNAs and miRNAs in plasma samples from patients with PCa and CTR and their respective ROC curves. **a** Expression analysis of patients *versus* controls. **b** Differential expression of *SIM2* and *OR51E2* genes in plasma samples from patients *versus* controls, both with PSA level ≤ 4.0 ng/mL and their respective ROC curves. **c** ROC curve referring to the score developed from the following transcripts: miR-200c, miR-200b, *OR51E2*, and *SIM2* genes; sensitivity of 67%, specificity of 75%. ROC = receiver operating characteristic, PCa = prostate cancer, CTR = controls subjects, PSA = prostate-specific antigen, AUC = area under the curve. *Statistically significant difference (Student’s *t*-test *P* < 0.05).

The analysis of cfmiRNA expression revealed differential expression of miR-200b (FC = -3.5, *P* = 0.02, AUC = 0.57) and miR-200c (FC = 1.9, *P* = 0.04, AUC = 0.62) (Fig 3A). No statistical differences were observed comparing PCa and control samples for the remaining six cfmiRNA.

To exclude the effect of age on gene expression, the mRNA/miRNA expression levels of cases and controls were compared in individuals below and above 65 years old (< 65 ys: 30 PCa and 26 controls; ≥65ys: 72 PCa and 24 controls). No significant differences were found according to the age (Table 3).

**Table 3:** Differential expression of circulating mRNAs and miRNAs in plasma samples from patients with PCa and controls based on age (< 65 and ≥ 65 years old).

	**miR-375**	**miR-143**	**miR-141**	**miR-200C**	**miR-183**	**miR-182**	**miR-200b**	**miR-133b**	**miR-133a**	**miR-205**	
age <65	0.04	7.87	-0.95	-0.42	0.14	4.71	4.56	0.76	0.37	-1.23	
age ≥ 65	0.37	7.61	0.03	0.32	-0.16	3.93	4.95	1.21	0.62	-1.92	
*P* value	0.3481	0.3993	0.2631	0.1004	0.4374	0.3246	0.6667	0.2171	0.4120	0.4737	
	***BCL2***	***NKX3*.*1***	***SIM2***	***PCA3***	***AMACR***	***OR5E21***	***TRPM8***	***GOLM1***	***COL1A1***	***FOX1A***	***MMP11***
age <65	-0.55	0.44	0.99	1.16	1.62	0.66	0.31	-3.51	5.19	1.39	-1.98
age ≥ 65	-0.56	0.40	1.51	1.31	2.14	1.91	0.89	-2.38	5.76	1.57	-1.62
*P* value	0.9818	0.9248	0.1841	0.7267	0.1848	0.1568	0.1748	0.3099	0.3591	0.5747	0.7050

*P* value was obtained by a T-test

An integrative analysis of the mRNAs and miRNAs differentially expressed was performed in the plasma from patients with PCa and controls. miR-200c, miR-200b, *OR51E2* and *SIM2* were used to construct a score to predict the risk of the disease. Altogether, these circulating markers showed sensitivity of 67% and specificity of 75% for PCa diagnosis (AUC = 0.71, *P* < 0.0001) (Fig 3B).

### Correlation of *OR51E2* and *SIM2* with clinical and histopathological parameters

Patients (n = 9) and controls (n = 50) with PSA ≤ 4.0 ng/μL, exhibited differential expression levels of *OR51E2* and *SIM2* genes, and the levels displayed by the patients were higher: *OR51E2* (FC = 47.92, *P* = 0.002, AUC = 0.79) and *SIM2* (FC = 5.21, *P* = 0.002, AUC = 0.85) (Fig 3C). *OR51E2* displayed 100% sensitivity and 50% specificity, while *SIM2* presented the same sensitivity but higher specificity (72%). No correlation was observed between expression levels of these genes and Gleason score (GS), bone metastasis, or bilateral tumor (*SIM2 P* = 0.49, P = 0.71, *P* = 0.36 and *OR51E2 P* = 0.78, *P* = 0.39, *P* = 0.38, respectively). Survival analysis could not be performed, owing to the short follow-up time of the patients.

### miR-200c and miR-200b as prognostic markers of PCa

**Fig 4:**
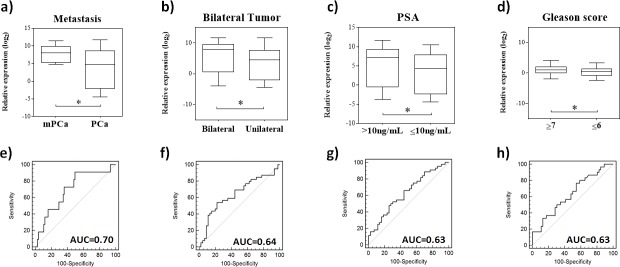
Comparison between circulating miR-200b expression levels and clinic-pathological features. The superior line represents the miR200b expression according to the: **a**. presence or absence of metastasis; **b**. unilateral or bilateral tumors; **c**. Low or high PSA levels; and, **d**. Gleason score ≤ 6 or ≥ 7. **e-h**. The ROC (Receiver Operating Characteristic) curve analysis for each histopathological feature. PCa = prostate cancer; mPCa = metastatic prostate cancer; AUC = area under the curve. *Statistically significant (Student’s t-test P < 0.05).

The expression analysis and ROC curve values of miRNAs associated with clinical and pathologic parameters are shown in Fig 4. miR-200b was overexpressed in patients with bone metastasis (FC = 12.4, *P* = 0.03, AUC = 0.70), bilateral tumor (FC = 5.22, *P* = 0.03, AUC = 0.64), and PSA level > 10.0 ng/μL (FC = 5.08, *P* = 0.03, AUC = 0.63) (Fig 4A and 4B).

The overexpression of miR-200c was directly proportional to the increase in the GS observed in PCa biopsies. Patients with GS = 7 exhibited miR-200c level twice higher than those detected in patients with GS ≤ 6 (P = 0.049). In addition, patients with GS = 8 were 4.8 times more likely to express miR-200c than those with GS ≤ 6 (P = 0.03). Patients with GS ≥ 7 showed 2.5 times more circulating miR-200c than those with GS ≤ 6 (P = 0.02, AUC = 0.63) (Fig 4).

## Discussion

Circulating nucleic acids are thought to be excellent biomarkers for the diagnosis of cancer, despite their technical limitation [14]. As reviewed by Rapisuwon et al. [41], one of the limitations in the cell-free mRNA evaluation is its relatively low abundance. In addition, mRNAs are subjected to degradation, instability, and intracellular mRNA contamination. For minimize these issues, the processing of plasma [30] and high-throughput protocols for cfRNA extractions should be performed, as we used in this study. Despite of these limitations, alteration in circulating RNA reflects dysregulation of cancer immunity, cell growth, proliferation and stromal interaction, which makes cfRNAs a suitable complementary tool to identify diagnostic and prognostic marker [14,41]. Currently, the application of cfmRNA in the diagnosis of PCa is poorly explored mainly by these limitations. In this study, we identified cfmRNAs and cfmiRNAs as potential biomarkers for the diagnosis and prognosis of PCa. The two cfmRNAs—*SIM2* and *OR51E2*—found to be overexpressed in plasma of patients with PCa, are known to play an important role in tumor biology as well as PCa development and progression [42,43].

The *SIM2* (single-minded 2) gene is a member of the family of transcription factors with *basic helix-loop-helix/per-Arnt-Sim* (bHLH/PAS) domains and has been involved in the pathogenesis of solid tumors [44,45]. In line with previous studies in prostate cell lines and tissues [42,46,47], the present study recorded an increase in the expression of *SIM2* in tumor tissues compared to the SNT (FC = 7.85, *P* < 0.001, FDR < 0.001). Arredouani et al. [46] reported the expression of SIM2 protein in the serum of patients with PCa and suggested its potential as a target for immunotherapy. Our data confirmed the involvement of *SIM2* gene in PCa.

In agreement with previous studies [48,49], *OR51E2* gene (olfactory receptor, family 51, subfamily E, member 2) exhibited differential expression levels in normal and tumor tissue samples (FC = 8.54, *P* < 0.001, FDR < 000.1). *OR51E2* is also known as a prostate-specific G-protein coupled receptor (*PSGR*) [48], and its *in vitro* inhibition retarded cell growth, suggests its potential as a target for cancer therapy [50]. The use of *OR51E2* as a non-invasive marker is poorly explored. Rigau et al. [51] suggested that *OR51E2* from urine sediment samples collected after prostate massage may be used as a biomarker for PCa screening. Similar results were also described by Sequeiros et al. [52] using similar sample types. In the present study, *OR51E2* overexpression was detected in circulating form in plasma of PCa patients.

To our knowledge, this is the first study showing differential expression of *SIM2* and *OR51E2* transcripts in the circulating form in plasma samples from patients and controls. These genes are potential biomarkers to be evaluated in plasma, allowing minimally invasive sample collection, easy detection, and wide application in clinical practice.

Approximately 15% of patients with PCa present PSA ≤ 4.0 ng/mL, making the diagnosis difficult [53]. In our study, only 9% of patients presented PSA ≤ 4.0 ng/mL. Despite this, our data showed that individuals with PSA level ≤ 4.0 ng/mL can be distinguished into cancer-free or PCa-affected based on the expression levels of *OR51E2* and *SIM2* genes. These two genes had sensitivity of 100% and specificity of 50% and 72%, respectively. We believe that the application of these genes as candidate diagnostic markers for PCa detection may fill the current gap in the diagnosis of PCa.

Among the miRNAs analyzed, miR-200c and miR-200b exhibited differential expression levels between patients and controls. Members of miR-200 family exert regulatory effect on genes involved in the epithelial-mesenchymal transition [54]. TGCA data analysis revealed that miR-200c was overexpressed in PCa tissues compared to the SNT, which is in agreement with other studies [55,56]. Our results are in line with those reported by Cheng et al. [55] wherein high levels of circulating miR-200c were detected in serum samples from patients with PCa compared to controls. In comparison to controls, patients with PCa exhibited a three-fold downregulation of miR-200b expression. Previous studies reported reduced miR-200b expression in PCa tissue samples and cell lines [57,40]. The miR-375 and miR-141 have been pointed out as diagnostic circulating biomarkers for PCa [58–61]. In agreement, our *in silico* analysis revealed the involvement of these miRNAs as candidate biomarkers. However, the plasma sample analysis showed no significant differences of miR-375 and miR-141 expression levels between patients and controls. Although not detected in our *in silico* analysis, several cfmiRNAs, including miR-21, miR-221 and miR-107 [62–67] have been reported as putative diagnostic markers in prostate cancer.

Association of *OR51E2* and *SIM2* genes with miR-200b and miR-200c as potential diagnostic markers for the disease was also investigated. Patients with PCa were discriminated from the cancer-free controls with a sensitivity of 67% and specificity of 75%. Salami et al. [3] showed that PSA test exhibits 80% sensitivity and 37% specificity for PCa diagnosis. The combination of four markers used in our study displayed better performance than the PSA test. We were unable to evaluate sensitivity and specificity of the PSA test in our population, owing to the limitation of cancer-free controls included in the experimental design. Nevertheless, the plasma markers *OR51E2* and *SIM2* showed the advantage over the PSA test to identify PCa patients with PSA ≤ 4.0 ng/mL.

A positive correlation was observed between miR-200c and GS; the higher expression levels of miR-200c, the higher the GS. According to Wu et al. [68] miR-200c is a good candidate marker for PCa detection. We found an association of miR-200c with bone metastasis, PSA level > 10.0 ng/μL, and bilateral tumor. In agreement with Wu et al. [68] and Bryant et al. [69], we suggested that this miRNA is a potential prognostic marker for PCa.

There is an unmet need for an improvement in the performance of PCa screening [20]. The present study showed that cfmRNAs and cfmiRNAs might be used as efficient diagnostic markers for PCa. In addition, miR-200b and miR-200c are prognostic marker candidates. Our study highlights the potential role of cfRNAs as efficient markers for complexes diseases. For the first time, the involvement of circulating RNA in PCa was reported, which can open avenues for a larger multicenter study to validate our results.

## Supporting information

S1 Table Primers sequence used in qPCR analysis for circulanting mRNA. (DOC)

S2 Table The 2267 differentially expressed genes in the *in silico*. (XLSX)

S3 Table Forty-nine differentially expressed miRNA in the *in silico* analysis. (XLSX)
