# Supplementary material for: Circulating mRNAs and miRNAs as candidate markers for the diagnosis and prognosis of prostate cancer
Source: PLoS One. 2017 Sep 14;12(9):e0184094. doi: 10.1371/journal.pone.0184094 (PMC5598937; doi:10.1371/journal.pone.0184094)
Supplement: S1 Table — (DOC) [file pone.0184094.s001.doc]

**Table S1.** Primers sequence used in qPCR analysis for circulanting mRNA

| **Gene** | **Primer Forward (5’-3’)** | **Primer Reverse (5’-3’)** | **Amplicon** | | |
| --- | --- | --- | --- | --- | --- |
|  |  |  | **Nucleotide position** | | **Size (bp)** |
|  |  |  | **Start** | **End** |  |
| *AMACR* | CCAAGGCTTATTTATGCCAG | CACCTGACAAAGCCAAATAG | 394 | 490 | 97 |
| *β-ACTIN* | CCCTCCATCGTCCACCGC | CTGCTGTCACCTTCACCGT | 1183 | 1382 | 200 |
| *GAPDH* | GGGCATCCTGGGCTACACT | GGTCCAGGGGTCTTACTC | 1001 | 1208 | 208 |
| *GOLM1* | ATGTCCTCCAGTTTCAGAAG | CTTCATTCCCCTTTTTGGTG | 616 | 756 | 141 |
| *MMP11* | GATAGACACCAATGAGATTGC | TTTGAAGAAAAAGAGCTCGC | 880 | 988 | 109 |
| *PCA3* | AGGTGAGAAATAAGAAAGGC | GTCATCTTGCTCTGTTTCTAGTG | 119 | 213 | 95 |
| *OR51E2* | CTGTATGGGCTCTACTGC | GCCACTACATACATGGAAAG | 182 | 354 | 173 |
| *SIM2* | GGATTTGTTTTTGTGGTAGC | ACCTGGGATAAGCCTAAATG | 894 | 976 | 83 |
| *TPRM8* | TGAGGAACAGAAGGAATGAC | CGCTTTCACTGTAAGACAAG | 69 | 158 | 90 |
| *FOX1A* | AGATGGAAGGGCATGAAACCA | GCCTGAGTTCATGTTGCTGAC | 165 | 259 | 95 |
